# Reversible Myocarditis and Pericarditis after Black Widow Spider Bite or Kounis Syndrome?

**DOI:** 10.1155/2015/768089

**Published:** 2015-10-05

**Authors:** Mehmet Yaman, Turkan Mete, Ismail Ozer, Elif Yaman, Osman Beton

**Affiliations:** ^1^Department of Cardiology, Samsun Training and Research Hospital, Ministry of Health, 55100 Samsun, Turkey; ^2^Department of Endocrinology and Metabolism, Samsun Training and Research Hospital, Ministry of Health, 55100 Samsun, Turkey; ^3^Department of Nephrology, Samsun Training and Research Hospital, Ministry of Health, 55100 Samsun, Turkey; ^4^Department of Pediatrics, Ondokuz Mayıs University Faculty of Medicine, 55100 Samsun, Turkey; ^5^Department of Cardiology, Faculty of Medicine, Cumhuriyet University, 58140 Sivas, Turkey

## Abstract

Clinical manifestation of black widow spider bite is variable and occasionally leads to death in rural areas. Cases of myocarditis and pericarditis after black widow spider bite are rare and the associated prognostic significance is unknown. Kounis syndrome has been defined as an acute coronary syndrome in the setting of allergic or hypersensitivity and anaphylactic or anaphylactoid insults that manifests as vasospastic angina or acute myocardial infarction or stent thrombosis. Allergic myocarditis is caused by myocardial inflammation triggered by infectious pathogens, toxic, ischemic, or mechanical injuries, such as drug-related inflammation and other immune reactions. A 15-year-old child was admitted to the emergency department with pulmonary edema after spider bite. ST segment depression on ECG, elevated cardiac enzymes and global left ventricular hypokinesia (with ejection fraction of 22%), and local pericardial effusion findings confirmed the diagnosis of myopericarditis. After heart failure and pulmonary edema oriented medical therapy, clinical status improved. Patient showed a progressive improvement and LV functions returned to normal on the sixth day. Myopericarditis complicating spider bite is rare and sometimes fatal. The mechanism is not clearly known. Alpha-latrotoxin of the black widow spider is mostly convicted in these cases. But allergy or hypersensitivity may play a role in myocardial damage.

## 1. Introduction

Black widow spider is a rare type of spider that generally lives in moderate climatic conditions and is rarely found in the rural area. It does not attack unless it is in confined places and disturbed [1]. It has very poisonous stings (arachnids) in the abdominal region. The clinical manifestations observed after spider bite are very variable and include abdominal pain, vomiting-nausea, headache, anxiety, itching, palpitations, and high blood pressure; however, pericarditis and myocarditis occur very rarely [2–7]. Our patient experienced myopericarditis resulting in pulmonary edema as well as muscle cramps, itching, urticarial lesions, anxiety, headache, palpitations, and high blood pressure.

## 2. Case Report

**Figure 1:**
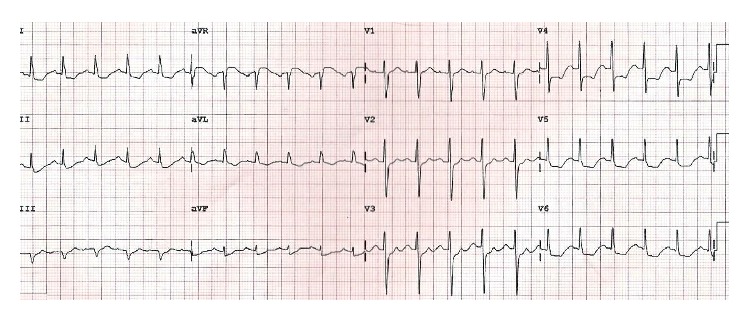
Admission ECG.

**Figure 2:**
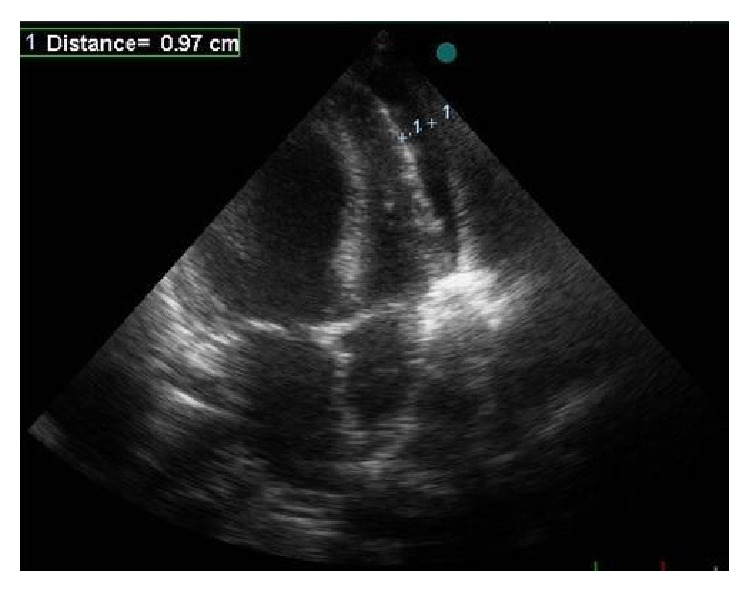
Pericardial fluid adjacent to the right ventricle.

A previously healthy 15-year-old male was admitted to the emergency department with acute respiratory depression and generalized pain. His relatives stated that the patient was bitten by a black widow spider approximately 5-6 hours ago. Vital signs were as follows: blood pressure: 160/100 mmHg, pulse: 124 beats/min, respiratory rate: 21 breaths/min, temperature: 37.3°C, and conscious: alert, cooperated, and agitated. There was a hyperemic lesion on the SCM muscle on the right side of the neck with a puncture on it. Heart sounds were found to be rhythmic and tachycardic, S1 and S2 were normal, and S3 was present on cardiac examination. Bilateral fine crackles were present up to upper zones on respiratory examination. Abdomen was sensitive with no defense or rebound on abdominal examination. Examinations of neurological and other systems were normal. The patient was taken to the intensive care unit with the diagnosis of pulmonary edema, rapidly put under monitoring, diuretics and nitroglycerine infusion started, and morphine ordered for the patient's pain. ECG revealed sinus tachycardia and ST segment depression at II, III, aVF, I, aVL, and V3–V6 leads (Figure 1). Telecardiography was consistent with cardiopulmonary edema. Mildly dilated left ventricle (LV), global hypokinesia of LV with ejection fraction (EF) of 22% (measured by modified Simpson's method), grade 1 diastolic dysfunction of LV, 1° mitral regurgitation, 35 mmHg of pulmonary artery systolic pressure, normal right ventricular dimensions, 16 cm of TAPSE, and pericardial fluid in the adjacency of the right ventricle (Figure 2) were detected on echocardiographic examination.

The laboratory results were as follows: leukocytes 23000 (normal range: 4–10 × 10^3^), mild elevation in the absolute eosinophil count (0.9 × 10^3^/L; normal range 0.0–0.4 × 10^3^/L), CRP: 59 mg/L (normal value: 0–8), CK: 280 (normal value: 55–170 u/L), CK-MB: 83 (<5 ng/mL), troponin I: 1.8 (<0.04 ng/mL), D-dimer: 8.7 (<250 ng/mL), and the other laboratory results were normal. The diagnosis was considered to be myopericarditis due to spider venom; medical treatment for acute heart failure was given for the first 2 days including iv diuretics and iv vasodilators. Then, medical treatment for chronic heart failure including ACEI, spironolactone, beta-blocker, and furosemide (oral) was given. Intermittent extremity contractions and urticaria like pruritic lesions in both gluteal regions were seen on the fourth day of the treatment. The cardiac enzymes were returned to the normal values and the echocardiography showed normal LV dimensions with EF of 62%, normal diastolic function, mild mitral regurgitation, and no pericardial effusion on the sixth day. The patient was discharged with beta-blocker and ACEI treatment. Medical treatment was stopped at 3rd month control, because he was asymptomatic and echocardiography was normal. After asymptomatic completion of year with quarterly period controls, annual control was scheduled for him and permission was given to his participation in school football team.

## 3. Discussion

Black widow spider is a member of the Arthropoda family and is commonly found worldwide. It generally lives in the Mediterranean Region [8], though our patient was exposed outside the Mediterranean region. Following snake and scorpion bites, myocarditis occurs commonly [9]; however, association of spider bite and myocarditis and pericarditis is very rare and may be fatal [3].

The main component of the black widow spider venom is alpha-latrotoxin, a toxin effective on the nervous system; it is a protein, which impacts the motor nerve endings, thereby leading to acetylcholine consumption and increased catecholamine release. While its primary target is the nervous system, sometimes it may affect organs such as the heart and the lungs [10, 11].

However, the underlying mechanism of myocarditis is not known. In a recent small meta-analysis by Dendane et al. [12] involving previous cases and a case of reversible myocarditis following black widow spider bite, hyperadrenergic state such as in broken-heart syndrome [13] is primarily claimed to be involved. Many authors also suggest that alpha-latrotoxin can result in direct myocardial injury [2–6]; there are cases of myocarditis occurring after black widow spider bite; however, pulmonary edema following myocarditis is rare [3, 6, 7, 12] and no cases of myocarditis and pericarditis have been reported. ST segment and T wave changes and increased cardiac markers have been reported in many cases [2–5].

Eosinophilia gave rise to question whether there is a role of allergic reaction on pathophysiology in this case. The type I variant of Kounis syndrome includes patients with normal coronary arteries without predisposing factors for coronary artery disease in whom the acute release of inflammatory mediators during the allergic reaction induces either coronary artery spasm without increase of cardiac enzymes or coronary artery spasm progressing to AMI with raised cardiac enzymes [14]. Hypersensitivity myocarditis is an inflammatory disease affecting the myocardial tissue and the cardiac conduction system manifesting mainly as a complication of drug therapy [14]. The only differences between these two hypersensitivity conditions are that in hypersensitivity myocarditis there is presence of eosinophils, atypical lymphocytes, and giant cells in myocardial biopsy, whereas biopsy in Kounis syndrome is typically normal [14]. Furthermore, the coronary angiogram in hypersensitivity myocarditis is normal, whereas angiogram in Kounis syndrome, especially in type II variant, shows the presence of coronary artery disease [14]. Mildly elevated cardiac enzymes and mild chest pain are characteristics of drug-induced myocarditis while eosinophilia may be absent in both conditions [14]. Hypersensitivity myocarditis can affect individuals of any age, but cases of Kounis syndrome have been reported in juveniles [14]. Physicians should be aware of the existence of hypersensitivity myocarditis and Kounis syndrome, because they are not rare diseases, but are very rarely diagnosed and very difficult to be differentiated diseases [14]. We did not perform neither coronary angiography nor myocardial biopsy for this patient, because relatively fast improvement in his clinic and his young age did not support the practicing of these procedures.

Myocarditis and allergic myocardial infarction known as the Kounis syndrome should be always kept in mind in patients with acute chest pain, ECG changes, and increased cardiac enzymes in case of normal coronary arteries [15]. In the Kounis syndrome, subendocardial layer of the myocardium is affected compatible with the territory of a particular coronary artery, while heterogeneous and subepicardial involvement are detected in acute myocarditis [15]. Cardiac magnetic resonance can differentiate acute myocarditis from acute myocardial infarction. In patients with myocarditis, late gadolinium enhancement is highly specific for diagnosing such injury in myocarditis [15]. In acute myocarditis, the distribution of gadolinium uptake is patchy and is not consistent with any coronary territory but usually appears in the subepicardial or mid-wall myocardial layers and never in the subendocardial area alone. In contrast, in acute myocardial infarction, the gadolinium late enhancement is always in the subendocardial area and consistent with the infarct-related artery. Late gadolinium enhancement not only allows differentiating ischemic injury, but may also indicate the possible etiology of the nonischemic insult [15]. We did not use MRI for confirmation of diagnosis, because cardiac MRI was not available in our hospital.

Besides direct toxin effect on myocardial damage, inflammatory response secondary to allergenic effect of toxin may have an additional role on myocardial damage causing either hypersensitivity myocarditis or Kounis syndrome type myocardial infarction. Further studies are needed to confirm this additional role, especially for prediction of the patients who will not benefit solely from classic medical treatment of acute heart failure and will need additional antiallergic treatment.

## 4. Conclusion

Myocarditis and pericarditis occurring after black widow spider bite are rare. Pulmonary edema and myocardial damage following myopericarditis complicating black widow bite were reversible in this case. Besides direct toxin effect on myocardial damage, inflammatory response secondary to allergenic effect of toxin may have an additional role on myocardial damage causing either hypersensitivity myocarditis or Kounis syndrome type myocardial infarction.

## References

[1] Gonzalez F. (2001). Black widow bites in children. *Journal of the American Osteopathic Association*.

[2] Sari I., Zengin S., Davutoglu V., Yildirim C., Gunay N. (2008). Myocarditis after black widow spider envenomation. *American Journal of Emergency Medicine*.

[3] Pneumatikos I. A., Galiatsou E., Goe D., Kitsakos A., Nakos G., Vougiouklakis T. G. (2003). Acute fatal toxic myocarditis after black widow spider envenomation. *Annals of Emergency Medicine*.

[4] Pulignano G., Del Sindaco D., Giovannini M. (1998). Myocardial damage after spider bite (*Latrodectus tredecimguttatus*) in a 16-year-old patient. *Giornale Italiano di Cardiologia*.

[5] Erdur B., Turkcuer I., Bukiran A., Kuru O., Varol I. (2007). Uncommon cardiovascular manifestations after a *Latrodectus* bite. *American Journal of Emergency Medicine*.

[6] Levine M., Canning J., Chase R., Ruha A. M. (2010). Cardiomyopathy following latrodectus envenomation. *The Western Journal of Emergency Medicine*.

[7] Bucur I. J., Obasi O. E. (1999). Spider bite envenomation in Al Baha region, Saudi Arabia. *Annals of Saudi Medicine*.

[8] Hoxha R. (2006). Two Albanians die from black widow spider bites. *British Medical Journal*.

[9] Wynne J., Braunwald E., Braunwald E., Zipes D. P., Libby P. (2001). The cardiomyopathies and myocardities. *Heart Disease: A Textbook of Cardiovascular Medicine*.

[10] Koh W. L. (1998). When to worry about spider bites: inaccurate diagnosis can have serious, even fatal, consequences. *Postgraduate Medicine*.

[11] Ichtchenko K., Bittner M. A., Krasnoperov V. (1999). A novel ubiquitously expressed *α*-latrotoxin receptor is a member of the CIRL family of G-protein-coupled receptors. *The Journal of Biological Chemistry*.

[12] Dendane T., Abidi K., Madani N. (2012). eversible myocarditis after black widow spider envenomation. *Case Reports in Medicine*.

[13] Wittstein I. S., Thiemann D. R., Lima J. A. C. (2005). Neurohumoral features of myocardial stunning due to sudden emotional stress. *The New England Journal of Medicine*.

[14] Kounis N. G., Mazarakis A., Tsigkas G., Giannopoulos S., Goudevenos J. (2011). Kounis syndrome: a new twist on an old disease. *Future Cardiology*.

[15] Almpanis G. C., Mazarakis A., Dimopoulos D. A. (2011). The conundrum of hypersensitivity cardiac disease: hypersensitivity myocarditis, acute hypersensitivity coronary syndrome (Kounis syndrome) or both?. *International Journal of Cardiology*.

